# Clinical Presentation and Management of Pulmonary Infection Caused by Mycobacterium paraffinicum

**DOI:** 10.7759/cureus.70813

**Published:** 2024-10-04

**Authors:** Rand Albahlawan, Mohamed Alafifi, Radoslaw Tereszkowski-Kaminski

**Affiliations:** 1 Internal Medicine, Stockport NHS Foundation Trust, Manchester, GBR

**Keywords:** antibiotic sensitivity, cavitating lung lesions, chronic cough, mycobacterium paraffinicum, non-tuberculous mycobacteria, ntm management, pulmonary infection

## Abstract

Non-tuberculous mycobacteria (NTM) are environmental organisms that rarely cause infections in healthy individuals but can be opportunistic pathogens in those with compromised immune systems or chronic lung conditions. *Mycobacterium paraffinicum*, a newly recognized species, is among these rare pathogens. We report a rare case of pulmonary infection with *M. paraffinicum*, detailing its clinical presentation, investigative findings, and treatment outcome. A 50-year-old male smoker with a history of chronic obstructive pulmonary disease (COPD), severe emphysema, and other comorbidities presented with chronic cough, progressive shortness of breath, and weight loss. Initial clinical suspicion focused on pulmonary tuberculosis (TB) due to his symptoms and family history, but sputum smear microscopy and subsequent biopsy revealed *M. paraffinicum*.

This diagnosis was confirmed by culture showing sensitivity to clarithromycin and amikacin and intermediate sensitivity to moxifloxacin. Investigations included chest X-ray and high-resolution computed tomography (HRCT), which revealed a large left apical cavity, progressive nodular wall thickening, and bilateral inflammatory nodules. The patient was initially managed as TB, but virology and serology screens were negative for common pathogens, including TB. The pivotal biopsy identified the NTM infection, leading to a revised diagnosis. Treatment was managed by a multidisciplinary team and included a regimen of rifampicin, ethambutol, azithromycin, and intravenous amikacin, alongside chest physiotherapy. Over a 12-month period, the patient showed significant clinical improvement, with reduced cough, improved appetite, and weight gain. Follow-up radiographs demonstrated notable improvement in the lung cavity. This case underscores the importance of considering NTM infections in differential diagnoses of cavitating lung lesions, particularly when initial treatments for TB are unsuccessful. Despite the lack of established treatment guidelines for *M. paraffinicum*, a combination of targeted antibiotics and supportive care proved effective. This case contributes to the limited literature on *M. paraffinicum*, highlighting the need for awareness and further research on management strategies for this rare pathogen.

## Introduction

Non-tuberculous mycobacteria (NTM) are a diverse group of environmental organisms commonly found in soil, water, and dust. While generally non-pathogenic in healthy individuals, NTMs can cause significant opportunistic infections, particularly in immunocompromised patients and those with underlying pulmonary conditions [1]. The Centers for Disease Control and Prevention (CDC) reports over 190 identified species of NTM [2], with increasing recognition of their role in pulmonary infections. In this case report, we present a rare case of *Mycobacterium paraffinicum* pulmonary infection. *M. paraffinicum* was initially described in the 1950s but only recognised as a distinct species in the 1990s following taxonomic re-evaluation [3]. The clinical manifestations, diagnostic challenges, and treatment of *M. paraffinicum* infections remain poorly understood due to the rarity of reported cases. This report aims to contribute to the limited body of knowledge by detailing the clinical course, diagnostic findings, radiological appearance and therapeutic approach, thereby enhancing understanding of this emerging pathogen within the context of pulmonary disease.

## Case presentation

A 50-year-old male smoker presented to the emergency department with worsening respiratory symptoms, including several years of progressively worsening shortness of breath, a productive cough with green phlegm streaked with blood, significant weight loss, and fatigue. He denied experiencing night sweats, fevers, or frank hemoptysis. His medical history included chronic obstructive pulmonary disease (COPD), severe emphysema, alcohol excess, previous venous thromboembolic disease, and epilepsy. His family history included a grandfather who had died of tuberculosis (TB). The patient had not received the Bacillus Calmette-Guérin (BCG) vaccine and had no history of recent travel or exposure to pets. On examination, he exhibited signs of respiratory failure, including tachypnea, increased work of breathing, and an oxygen saturation of 83%. Cardiovascular signs were stable. Auscultation of the chest revealed reduced air entry in the left upper lobe and a mild scattered wheeze, but no palpable lymph nodes or other significant clinical findings.

**Figure 1:**
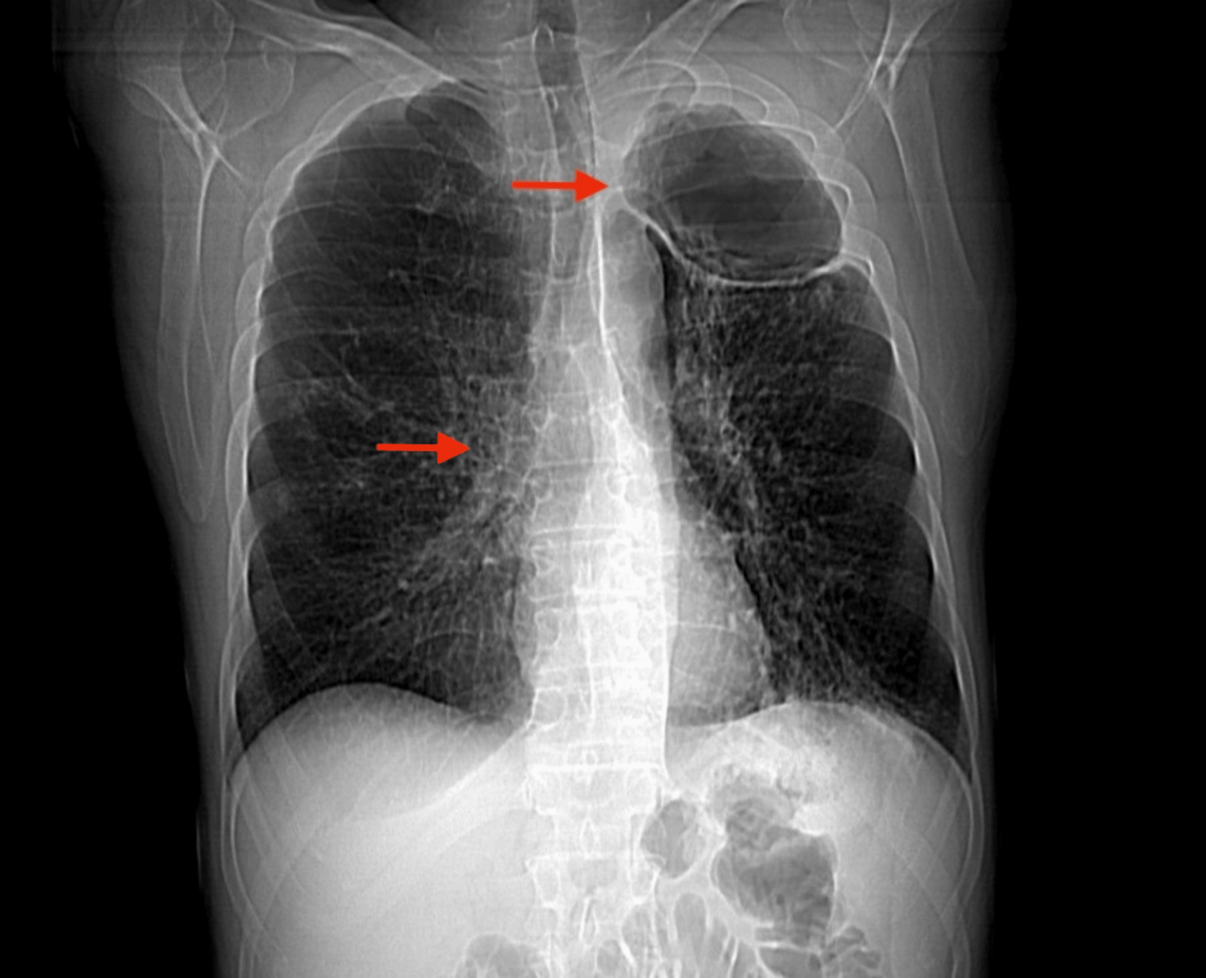
Chest AP erect plain film Chest X-ray with an arrow highlighting a large left apical cavity with nodular wall thickening. A second arrow points to several perihilar and infrahilar nodules.

**Figure 2:**
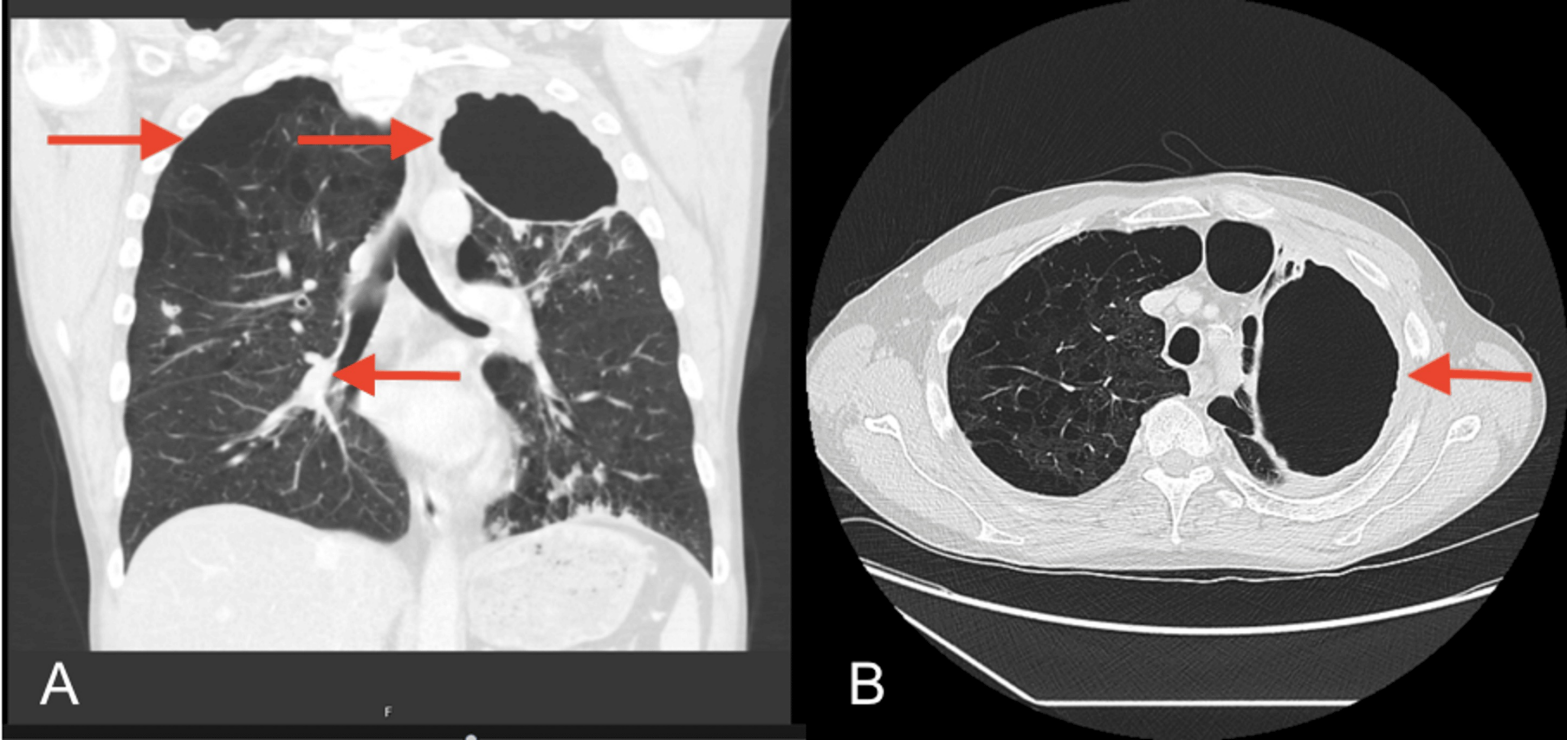
Computed tomography (CT) scan Image A highlights a large chronic cavitating lesion in the left upper lobe, marked by an arrow, with progressive nodular wall thickening. Additional arrows point to bilateral inflammatory nodules, tree-in-bud changes in both upper lobes, and bullous emphysema. Image B presents a cross-sectional view of the same pathology, with an arrow indicating the large apical cavitating lesion.

**Figure 3:**
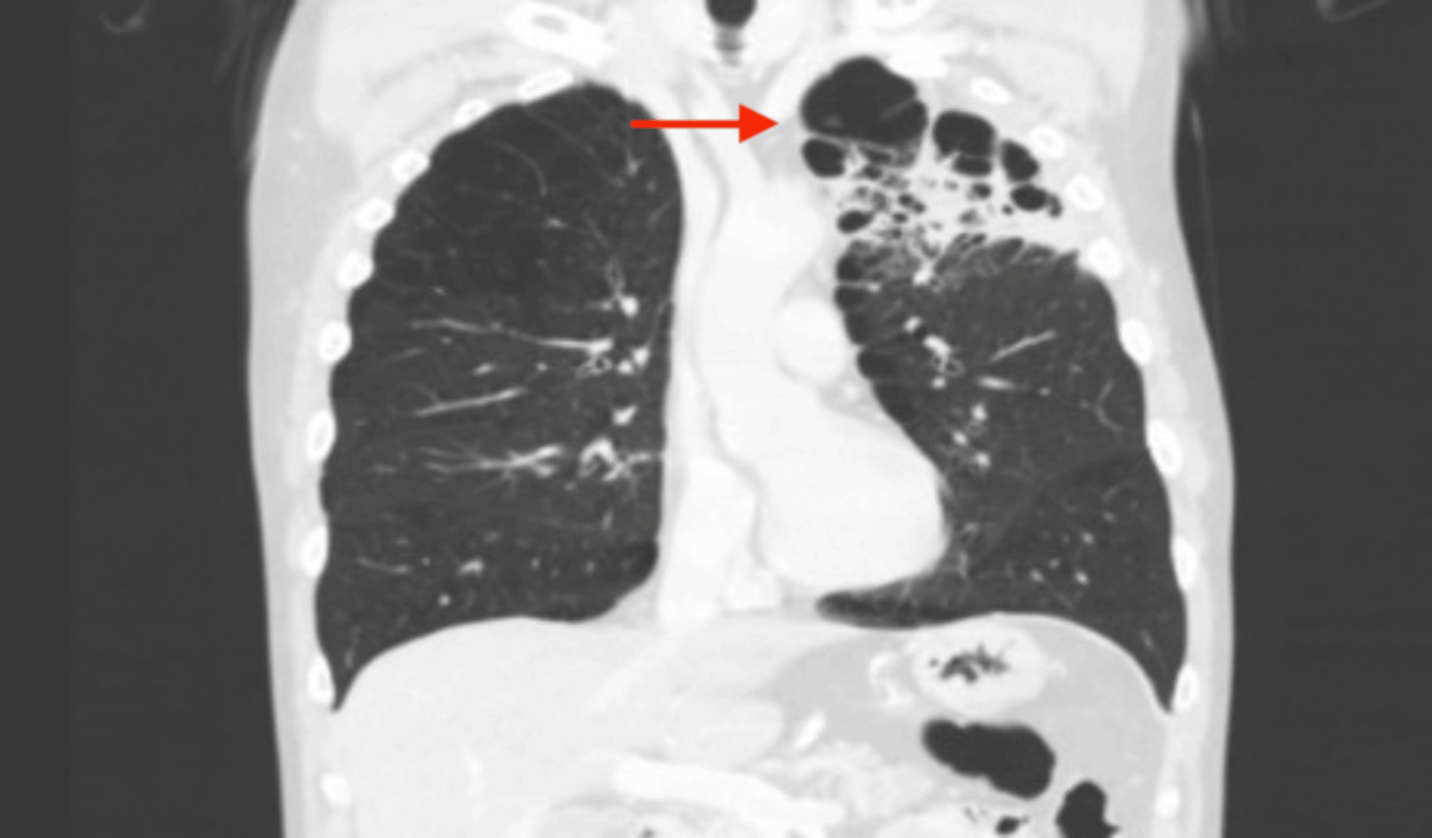
Computed tomography (CT) scan of the thorax An arrow highlights an early-stage cavity, initially presenting as inflammatory changes in the left upper lobe, with fluid accumulation visible within an emphysematous bulla.

Initial investigations included a chest X-ray which revealed a large left apical cavity with wall thickening, along with several perihilar and infrahilar nodules (Figure 1). A CT scan of the thorax showed a chronic cavitating lesion in the left upper lobe with progressive nodular wall thickening and worsening bilateral inflammatory nodules (Figure 2). Additional findings included cavitating nodules, tree-in-bud changes in both upper lobes and bullous emphysema. A review of previous imaging indicated that inflammatory changes in the left upper lobe had begun as fluid within emphysematous bulla two years earlier, which had progressed over time into a large thick-walled cavity (Figure 3).

**Figure 4:**
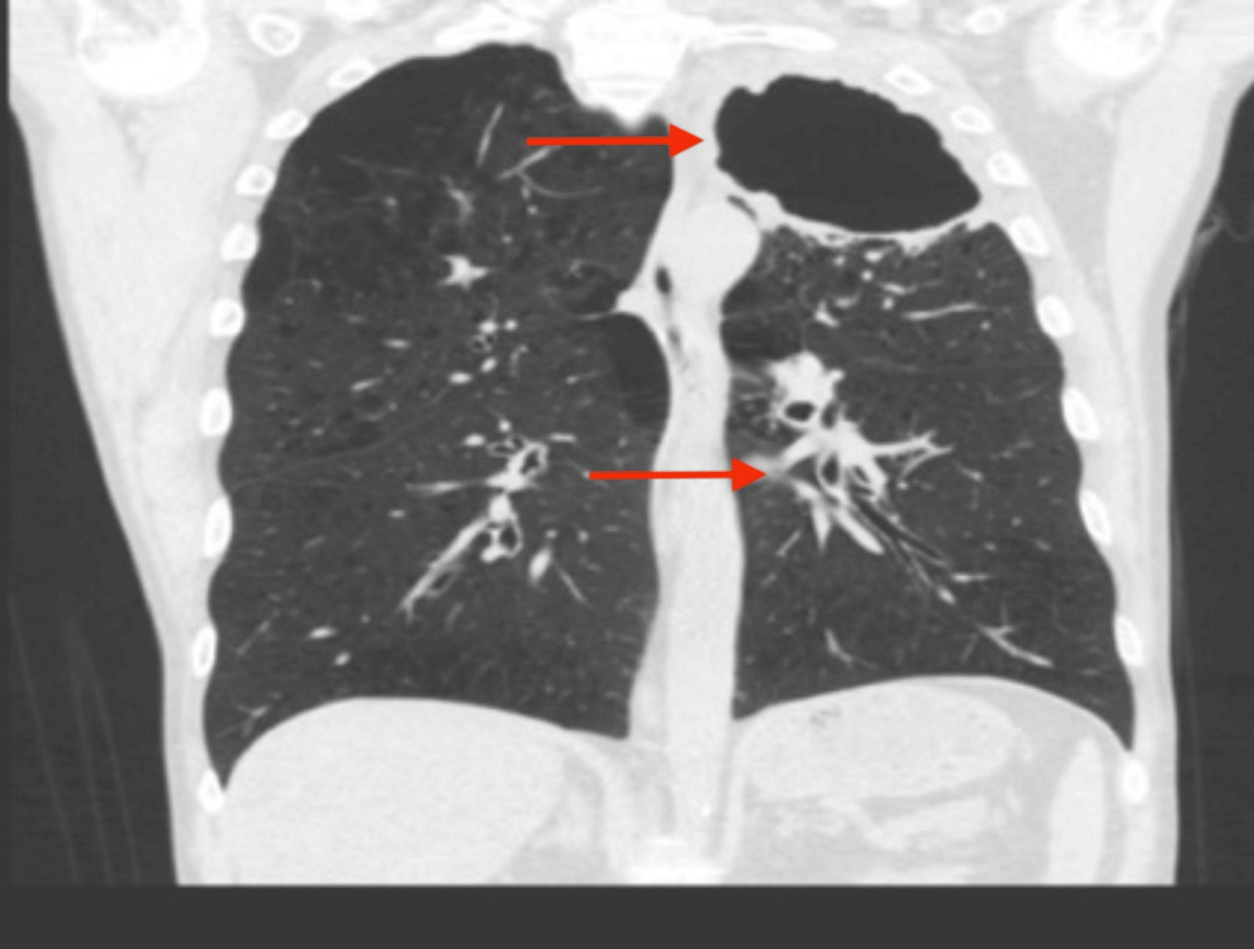
High-resolution computed tomography (HRCT) of the thorax Arrows point to evidence of disease progression, including the development of a thick-walled cavity in the left apex and multifocal solid nodules in the left upper lobe.

Serological tests revealed raised infectious and inflammatory markers, while a virology screen for HIV 1+2, hepatitis C antibody, and hepatitis B surface antigen was negative. Although total IgE levels were elevated (447 kU/L), allergic bronchopulmonary aspergillosis (ABPA) was ruled out, as both IgE and IgG levels against Aspergillus fumigatus were <0.1 kU/L. High-resolution computed tomography (HRCT) confirmed progressive disease with the development of a thick-walled left apical cavity and multifocal solid nodules in the left upper lobe (Figure 4). Sputum smear microscopy was positive for acid-fast bacilli. Given the family history of TB, radiological findings, and the patient's history of chronic chest symptoms and weight loss, the initial clinical impression was pulmonary TB. However, an ultrasound-guided biopsy of the cavity's internal walls proved pivotal, as it provided samples that led to the identification of NTM, later confirmed as *M. paraffinicum*. Culture revealed sensitivity to clarithromycin and amikacin and intermediate sensitivity to moxifloxacin.

**Figure 5:**
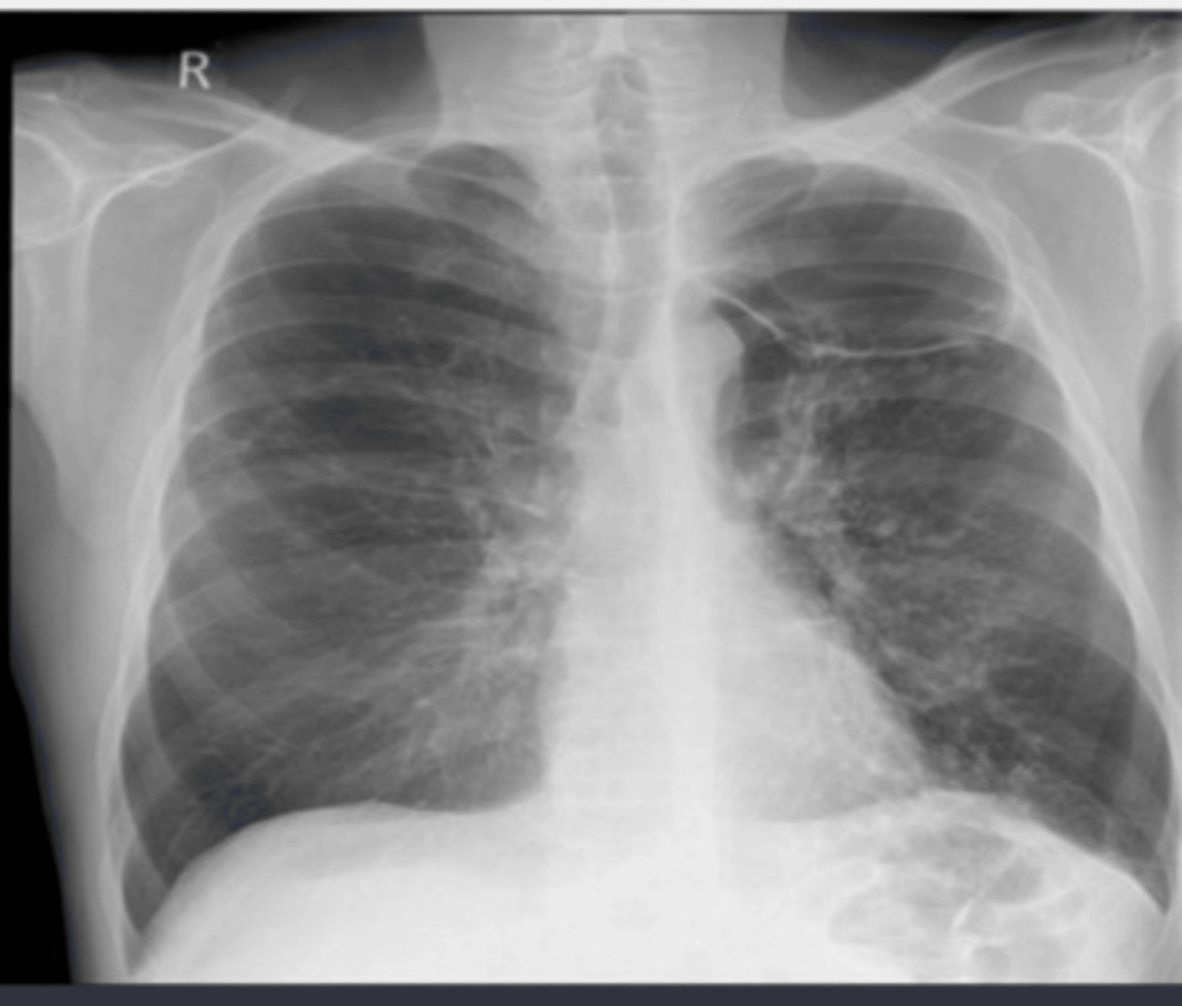
Chest AP erect plain film The image shows significant improvement in the appearance of the lung cavity.

The patient was referred to a tertiary infectious disease centre for multidisciplinary management. Baseline assessments, including blood tests, ECG, vision checks, and audiology, were all normal. He was started on a 12-month course of rifampicin (600 mg daily), ethambutol (1100 mg daily), and azithromycin (250 mg daily). In addition, he received intravenous amikacin (1.5 g three times weekly) for 12 weeks through the Outpatient Parenteral Antimicrobial Therapy (OPAT) service. Regular chest physiotherapy was provided for airway clearance. At the two-month follow-up, the patient reported good tolerance to the antibiotic regimen, with minimal side effects. He noted improvements in his cough, reduced production of purulent sputum, and increased appetite and weight. Monitoring for potential side effects included regular blood tests (full blood count, liver function, renal function), ECG, and amikacin levels, all of which remained stable. A follow-up chest X-ray at three months showed significant improvement in the appearance of the lung cavity (Figure 5).

## Discussion

*M. paraffinicum* is a slow-growing, strongly acid-fast, non-tuberculous rod first isolated from oil field soils in the 1950s [4]. Initially, this organism was thought to be indistinguishable from *M. scrofulaceum* due to similar morphological and biochemical characteristics. As a result, by the 1970s, it was removed from the mycobacterial taxonomy. However, further advances in molecular techniques and phylogenetic studies in 1991 led to the reclassification of *M. paraffinicum* as a distinct species [3]. To date, very few cases of *M. paraffinicum* as the causative pathogen in symptomatic human infections have been reported, with our case being one of these rare instances. In addition to these rare cases, there have been reports of *M. paraffinicum* being isolated incidentally from sputum samples of patients undergoing treatment for other conditions [5,6]. These incidental findings highlight the organism’s possible presence as a coloniser rather than an active pathogen. However, for this discussion, we will focus solely on cases where *M. paraffinicum* was identified as the primary cause of symptomatic disease.

In terms of demographics for the reported case studies, four cases were identified: two in women and two in men [4,7-9]. Three of these cases involved individuals over the age of 80, while only one case affected a patient in their 60s. The pulmonary pathology associated with *M. paraffinicum* infection has shown some consistent features. Including the current case, cavitating lung disease was observed in three of the five total cases, indicating that cavitation may be a characteristic finding in this rare infection [7,8]. Pulmonary nodules were also a common finding on CT imaging in all four patients, suggesting a trend of nodular disease [4,7,8]. One notable case described by Tan and Perera involved a patient who presented with cervical lymphadenitis and minimal respiratory symptoms apart from a mild chronic cough, illustrating that this bacterium can manifest in non-pulmonary forms as well [9]. Management of NTM infections, including those caused by *M. paraffinicum*, remains a clinical challenge due to the lack of established treatment guidelines [10]. The rarity of *M. paraffinicum* infections makes standardized treatment approaches even more elusive. Antibiotic regimens tailored to NTM infections are typically based on susceptibility profiles, but treatment outcomes for *M. paraffinicum* remain under-researched. In two documented cases, treatment had to be discontinued due to side effects, and the patients were subsequently lost to follow-up [4,8]. Despite these challenges, Barretto et al. reported a favourable clinical response to oral azithromycin administered three times a week, with the patient showing improvement at a three-month follow-up. However, it is important to note that despite clinical improvement, the patient’s acid-fast smear remained positive, suggesting that the infection was not fully eradicated [8]. The most recent case, described by Collazo-Santiago et al., involved an inpatient regimen of imipenem, amikacin, and azithromycin, which resulted in sufficient improvement for hospital discharge after approximately one month [7]. Upon discharge, the patient continued treatment with azithromycin and ciprofloxacin, and a follow-up CT scan at three months showed marked radiological improvement, indicating successful management of the infection. In the case where *M. paraffinicum* caused lymphadenitis, the clinical course was somewhat different [9]. The infected mass discharged spontaneously and healed without the need for immediate antibiotic treatment. This spontaneous resolution suggests that in some cases, *M. paraffinicum* infections may have a self-limiting nature, particularly in non-pulmonary forms of the disease. However, given the small number of reported cases, further research is needed to fully understand the spectrum of disease caused by this rare pathogen and to develop evidence-based treatment strategies.

## Conclusions

This case report highlights a rare instance of *M. paraffinicum* infection, adding to the limited number of reported cases worldwide. Despite its initial identification over half a century ago, *M. paraffinicum* remains an exceedingly rare pathogen, with few cases of symptomatic infection and limited clinical data available for guiding management. Our case further emphasizes the need for heightened clinical suspicion in immunocompromised patients presenting with chronic pulmonary symptoms, especially when conventional diagnoses, such as TB, are initially favoured but proven negative. The clinical presentation of *M. paraffinicum* infection appears to involve cavitating lung lesions, nodules, and in some cases, bronchiectasis, all common features of NTM infections. Despite these recurring radiological findings, management remains challenging due to the lack of standardized treatment protocols.

The outcome of our patient, along with prior reported cases, suggests that while antibiotic regimens may lead to clinical improvement, persistent colonisation and relapse are concerns that warrant long-term follow-up. Furthermore, the variable response to treatment across cases highlights the importance of individualized patient care and the potential for side-effect-driven treatment discontinuation. As NTM infections become more recognised, especially in ageing and immunosuppressed populations, it is crucial to expand the research and clinical understanding of rare species like *M. paraffinicum*. More cases and studies are needed to establish effective treatment strategies, improve patient outcomes, and better understand the epidemiology of this elusive pathogen.
